# Silica Vesicle Nanovaccine Formulations Stimulate Long-Term Immune Responses to the *Bovine Viral Diarrhoea* Virus E2 Protein

**DOI:** 10.1371/journal.pone.0143507

**Published:** 2015-12-02

**Authors:** Karishma T. Mody, Donna Mahony, Antonino S. Cavallaro, Jun Zhang, Bing Zhang, Timothy J. Mahony, Chengzhong Yu, Neena Mitter

**Affiliations:** 1 Queensland Alliance for Agriculture and Food Innovation, The University of Queensland, Brisbane, Australia; 2 Australian Institute for Bioengineering and Nanotechnology, The University of Queensland, Brisbane, Australia; 3 Animal Science, Queensland Department of Agriculture and Fisheries, Brisbane, Australia; University of Nebraska-Lincoln, UNITED STATES

## Abstract

Bovine Viral Diarrhoea Virus (BVDV) is one of the most serious pathogen, which causes tremendous economic loss to the cattle industry worldwide, meriting the development of improved subunit vaccines. Structural glycoprotein E2 is reported to be a major immunogenic determinant of BVDV virion. We have developed a novel hollow silica vesicles (SV) based platform to administer BVDV-1 *Escherichia coli*-expressed optimised E2 (oE2) antigen as a nanovaccine formulation. The SV-140 vesicles (diameter 50 nm, wall thickness 6 nm, perforated by pores of entrance size 16 nm and total pore volume of 0.934 cm^3^g^-1^) have proven to be ideal candidates to load oE2 antigen and generate immune response. The current study for the first time demonstrates the ability of freeze-dried (FD) as well as non-FD oE2/SV140 nanovaccine formulation to induce long-term balanced antibody and cell mediated memory responses for at least 6 months with a shortened dosing regimen of two doses in small animal model. The *in vivo* ability of oE2 (100 μg)/SV-140 (500 μg) and FD oE2 (100 μg)/SV-140 (500 μg) to induce long-term immunity was compared to immunisation with oE2 (100 μg) together with the conventional adjuvant Quil-A from the *Quillaja saponira* (10 μg) in mice. The oE2/SV-140 as well as the FD oE2/SV-140 nanovaccine generated oE2-specific antibody and cell mediated responses for up to six months post the final second immunisation. Significantly, the cell-mediated responses were consistently high in mice immunised with oE2/SV-140 (1,500 SFU/million cells) at the six-month time point. Histopathology studies showed no morphological changes at the site of injection or in the different organs harvested from the mice immunised with 500 μg SV-140 nanovaccine compared to the unimmunised control. The platform has the potential for developing single dose vaccines without the requirement of cold chain storage for veterinary and human applications.

## Introduction

Development of veterinary vaccine comes with a spectrum of challenges, as the storage, shipping and administration of the vaccine should be easy and the cost of veterinary vaccine production needs to be kept low. [1] Subunit vaccines often need to be refrigerated, require addition of adjuvants and need to be administered multiple times in order to induce long-term immunity. Therefore, to reduce the need for booster immunisations, a system needs to be developed that delivers the antigen and also acts as an efficient adjuvant. Adjuvants are biomolecules that are added to vaccines to stimulate immune responses, however, only a few adjuvants have been approved for human as well as veterinary use. [2] Another issue that needs to be taken into account is the transport and storage of subunit vaccines that require cold chain storage, which can be challenging and expensive, especially in remote areas. The storage stability of the subunit vaccines can be improved by freeze-drying the vaccine formulations. Excipients such as sugars, surfactants, amino acids and polymers are typically added to the vaccine formulations to prevent degradation in the freeze-drying process and aide in the reconstitution of vaccines prior to use. [3,4]

Bovine viral diarrhoea virus 1 (BVDV-1) is a single-stranded RNA virus that is a major contributor to the bovine respiratory disease complex and other disease of cattle. BVDV-1 infection of cattle has been highly investigated in several countries as it causes tremendous economic losses to the cattle industry. [5] The major source of new BVDV-1 infection in herds come from the secretions or body fluids of persistently infected or acutely infected animals. [6] Immunosuppression caused by BVDV-1 infection can lead to a secondary infection, which is a major cause of death in BVDV-1 infected cattle. [7] The current BVDV-1 vaccine approved for use in Australia needs to be refrigerated at 2°C to 8°C.

The structural protein, E2, from BVDV is a major immunogenic determinant and is an ideal candidate as a subunit vaccine as it can elicit neutralising antibodies. [8] BVDV species have been classified into type-1 and type-2 viruses, [9,10] and acute infections with BVDV-1 isolates are often subclinical [11], whereas acute infections with BVDV-2 often result in severe clinical signs with high mortality. [12] In a recent study, a plant expressed truncated version of BVDV E2 fused to a recombinant single chain antibody (scFv), named APCH was used to target antigen presenting cells and the APCH-tE2 was found to induce neutralising antibodies in bovines. [1] Further, BVDV E2 formulated with a combination adjuvant poly[di(sodium carboxylatoethylphenoxy)]-phosphazene, a toll-like receptor agonist and an innate defence regulator peptide (designated as TriAdj) induced antibody and cell-mediated immune responses and provided protection in animals from BVDV-2 infection. [9]

The role of mesoporous silica nanoparticles (MSNs), as antigen carriers and self-adjuvant vaccines has been investigated to develop successful vaccine delivery systems targeting BVDV-1. [13,14,15,16] In addition, we have also developed freeze-dried (FD) silica nanoparticle based vaccine delivery systems and have demonstrated that the model protein ovalbumin (OVA) adsorbed mesoporous silica nanoparticles can be freeze-dried using 5% (w/v) trehalose and 1% PEG8000 (w/v) as excipients. [13] The FD 10 μg OVA/150 μg AM-41 formulation was tested in mice and the nanovaccine stimulated both antibody and cell-mediated immune responses. [13,16] Amino functionalised hollow mesoporous silica nanoparticles (HMSAs) with small pore entrance size 2 to 3.5 nm [15] were trialled for adsorption of BVDV codon optimised E2 antigen expressed in *E*. *coli* (oE2) (80 mg oE2 bound to per 1 g of HMSA). Although this formulation elicited antibody as well as cell mediated responses after three injections, the constraint here was the low antigen binding capacity of the HMSA particles. [15] To address this issue and in order to move towards our goal of generating enhanced immune response in comparison to conventional adjuvant Quil-A, we designed novel silica vesicles (SV) of ~ 50 nm with a thin shell wall of 6 nm and controlled entrance size (varying between 5.7 nm to 16 nm). These SV exhibited higher loading to Ribonuclease A with sustained release behaviour. [17] The SV-140 with an entrance size of 16 nm significantly improved the oE2 adsorption; ~250 mg oE2 bound to per g SV. The oE2 (50 μg)/SV-140 (250 μg) induced anti-oE2 IgG (10^5^) and interferon-γ (IFN-γ) responses stronger than the conventional adjuvant Quil-A (anti-oE2 IgG response of 10^4^) after three subcutaneous injections. [14]

To further develop SVs as vaccine nanocarriers which are self-adjuvanting, the first goal of the current study was to determine if the oE2/SV140 formulation previously shown to be effective in generating antibody and cell-mediated response can also stimulate longer term immune responses with reduced number of injections. The second goal was to develop FD oE2/SV140 formulation and compare it with oE2/SV140 for addressing the issue of storability. In this work for the first time we have demonstrated that vaccination with oE2 (100 μg)/SV-140 (500 μg) elicited antibody and cell-mediated responses not only after three weeks of two subcutaneous injections but also showed strong cell-mediated responses for at least six months after the second immunisation. In addition, we have also shown that FD oE2/SV-140 nano-formulation also induced both antibody and cell-mediated immune responses. This work for the first time clearly demonstrates the potential of silica vesicles for developing a nanovaccine with reduced number of injections, induction of long-term immune responses and improved storage.

## Materials and Methods

### Freeze-drying process

The SV-140 were prepared and adsorption reactions were set up as previously described. [14] The oE2 adsorbed SV-140 samples were centrifuged at 16.2 *g* for 5 min and the supernatants were removed. Prior to freeze-drying, oE2/SV-140 pellets were resuspended in different combinations and concentrations of excipients (S1 Table). Samples were frozen in liquid nitrogen then placed in a freeze-dryer (Martin Christ Model LPC-32, Osterode AM Harz, Germany) at 24°C, 0.11 mbar for 22 h for drying. Freeze-dried samples were stored in a vacuum desiccator at ambient temperature (25°C). The optimal excipients trehalose (5% final concentration) and glycine (0.1% final concentration) were added to the oE2 bound vesicles and the final volume adjusted to 1 ml. The oE2 (500 μg) plus Quil-A (2mg/mL) sample was prepared in sterile injectable water without any excipients and the SV-140 alone with excipients 5% trehalose and 0.1% glycine. All the samples were freeze-dried as described in this section.

### Western hybridisation

Following SDS-PAGE electrophoresis the oE2 protein in the nanovaccine formulations was detected using Western blot hybridisation as previously described [15] using mouse anti-E2 sera at 1:4000 dilution. The secondary antibody anti-mouse Immunoglobulin G HRP conjugate (Chemicon, Millipore, Billerica, Massachusetts, USA) was used at 1:2,000 dilution and anti-bovine Immunoglobulin G HRP conjugate (Zymed) at 1:10,000 dilution. Detection was carried out using an ECL detection kit (GE Healthcare).

### Reconstitution and transmission electron microscope (TEM) and scanning electron microscope (SEM) of lyophilised samples

Samples were reconstituted in 1 mL water. The physical characteristics of the freeze-dried vesicles in solution were observed using transmission electron microscopy (TEM) and scanning electron microscopy (SEM) before and after reconstitution the freeze-drying preparations.

### Ethics Statement

Mice were euthanised according to the ethics by CO2 inhalation. All procedures were approved by The University of Queensland, Ethics Committee, (Approval No: 2012001137) as required by the Animal Care and Protection Act (2001) and The Australian Code for the Care and Use of Animals for Scientific Purposes (8^th^ Edition). [18]

### Immunisation studies conducted in mice

C57BL/6J mice were purchased from and housed in the Biological Resource Facility, The University of Queensland, Brisbane, Australia under specific pathogen-free conditions. Eight week old female mice were housed in HEPA-filtered cages with eight animals per group in an environmentally controlled area with a cycle of 12 h of light and 12 h of darkness. Food and water were given *ad libitum*.

Pre-immunisation (PI) blood samples were collected prior to the first injection and processed as detailed in [14]. The conventional adjuvant Quil-A (Superfos Biosector, Vedback, Denmark) was resuspended at 2 mg/mL in sterile injectable water (Pfizer, Brooklyn, USA). The non-FD and FD nanovaccines were prepared by adsorbing oE2 to SV-140 as described above and in [14], the positive control was oE2 (100 μg) plus Quil-A (10 μg) or FD oE2 (100 μg) plus Quil-A (10 μg). The negative control was prepared by freeze-drying SV-140 alone with 5% trehalose plus 0.1% glycine. The vaccines were administered to investigate the induction of immune responses and the treatment groups received injections as mentioned in Table 1. Two injections were administered at 3 week intervals to all the treatment groups except for the unimmunised group. Dose volumes of 100 μL (in 0.9% saline, Pfizer) were administered by subcutaneous injection at the tail base using a sterile 27 gauge needle (Terumo, Tokyo, Japan). Four mice from each group were sacrificed 21 days after the final immunisation. Blood samples from the remaining four mice were collected every four weeks via tail bleeds for up to six months and at the end of the trial period animals were sacrificed. The animals were weighed and monitored for general health once a week. All the animals were assessed weekly and they remained in good health throughout the duration of the study.

**Table 1:** Immunisation groups in mice trial. All doses were administered subcutaneously at the tail base.

Group	Prototype vaccine/Injection Dose
1	oE2 (100 μg) + QuilA (10 μg)
2	FD oE2 (100 μg) + Quil A (10 μg)
3	oE2 (100 μg) / SV-140 (500 μg)
4	FD oE2 (100 μg) / SV-140 (500 μg)
5	FD SV-140 (500 μg)
6	Unimmunised

### Enzyme-Linked ImmunoSorbent Assay (ELISA) protocol

The detection of oE2-specific antibodies were performed by coating microtitre plates (96 well, Nunc, Maxisorb, Roskilde, Denmark) with oE2 antigen solution (2 ng/μL, 50 μL) in PBS overnight at 4°C. The coating solution was removed and the plates were washed once with PBS-T (1x PBS, 0.1% Tween-20, Sigma-Aldrich) and blocked with Bovine Serum Albumin (5%, Sigma-Aldrich) and skim milk (5%, Fonterra, Auckland, New Zealand) in 200 μL PBS for 1 h with gentle shaking at RT. Plates were washed three times with PBS-T.

Mouse sera samples were diluted from 1:100 to 1:6400 in 50 μL PBS and each dilution was added to the wells of the blocked plates followed by incubation for 2 h at RT. To detect mouse antibodies HRP conjugated polyclonal sheep anti-mouse IgG antibodies (Chemicon Australia, Melbourne, VIC, Australia) diluted in PBS to 1:50,000 were added to each well and incubated for 1 h at RT with gentle shaking. Plates were washed three times in PBS-T. TMB substrate (100 μL, Life Technologies) was added to each well and incubated for 15 min at RT; 100 μL of 1N HCl was added to the wells to stop the chromogenic reaction. The plates were read at 450 nm on the BioTek microplate reader (Winooski, US).

### Isolation of murine splenocytes and enzyme-linked immunosorbent spot (ELISPOT) Assay

Spleens were aseptically removed following euthanasia from the four animals sacrificed at three weeks and the other four at six months after the final immunisation; the collected spleens were processed as previously described. [14]

Cells from each mouse spleen were seeded at 1.0–1.5 x 10^5^ cells/well in triplicate into Polyvinylidene fluoride (PVDF) ELISPOT plates precoated with monoclonal interferon- γ (IFN-γ) (Mabtech, Sweden) capture antibody. Cells were incubated in complete DMEM medium at 37°C and 5% CO_2_ for 40 h in the presence or absence of 1 μg/mL oE2 antigen or the polyclonal activator concavalin A (Con A, 1 μg/mL, Sigma Aldrich) as a positive control. IFN-γ ELISPOT assays were performed according to the manufacturer’s specifications. The ELISPOT plates were read on an ELISPOT reader (Autoimmun Diagnostika, Strassburg, Germany).

### Immunohistochemistry (IHC)

Spleen sections were collected from the sacrificed mice at both the time points three weeks and six months. A part of the spleen was dissected and frozen in OCT embedding medium and 5 μm sections were cut using Hyrax C60 cryostat. The slides with cryosections were fixed in cold ethanol on ice for 8 min and then dried at RT for 20 min. The slides were then washed 3 x 5 min in PBS, left to dry at RT for 20 min and using a Dako pen circles were marked around the sections. The sections were then incubated overnight with the blocking buffer (PBS containing 1% BSA and 5% FBS) at 4°C. Next day, to remove the block the slides were washed for 5 min in PBS three times. The sections were then incubated with Alexa Fluor 488 Goat Anti-Mouse IgG at 1:500 for 1 h at RT in dark, the slides were then washed as previously described in PBS. To stain the nuclei of cells the sections were then incubated with DAPI for 5 min and quickly washed in PBS. The sections were mounted with ProLong® Gold Antifade mounting medium and examined under the microscope.

### Histopathology

Heart, kidney, liver and injection sites from the sacrificed mice were collected and fixed in 10% formalin for 48 h. The organs were further processed and embedded in paraffin and 8 μm sections were cut using the Leica RM 2245 Rotary Microtome. The sections were then stained using the following haematoxylin and eosin staining procedure. Sections were first Dewaxed in xylene (3 changes of 2 min each), and then rehydrated in absolute alcohol (2 changes of 2 min each), in 90% for 2 min, in 70% for 2 min. Then washed in running tap water for 2 min and stained in haematoxylin for 3 min and again washed in running tap water for 2 min. Sections were then washed in 70% alcohol for 2 min and stained in eosin for 3 min. Sections were then washed in 95% alcohol for 2 min, then in absolute alcohol (3 changes of 2 min each). Finally, the sections were rapidly dehydrated and fixed in xylene (3 changes of 2 min each) and mounted in DePeX. The sections were then observed under the Zeiss LSM 510 META confocal microscope.

### Statistical analyses

Statistical analysis of the ELISA data was performed on the average OD values (at 450 nm) of individual animals in each group (serum dilution of 1:1600). The ELISA results were analysed by one-way analysis of variance and significant differences between groups were determined using Tukey’s HSD test (GraphPad Prism for Windows V5.04).

Statistical analysis of the ELISPOT data was performed on the number of SFU/million cells obtained for individual animals using an unpaired, two-tailed Student’s t-test (Microsoft Excel).

## Results

### Physicochemical properties of FD oE2 SV-140

**Fig 1:**
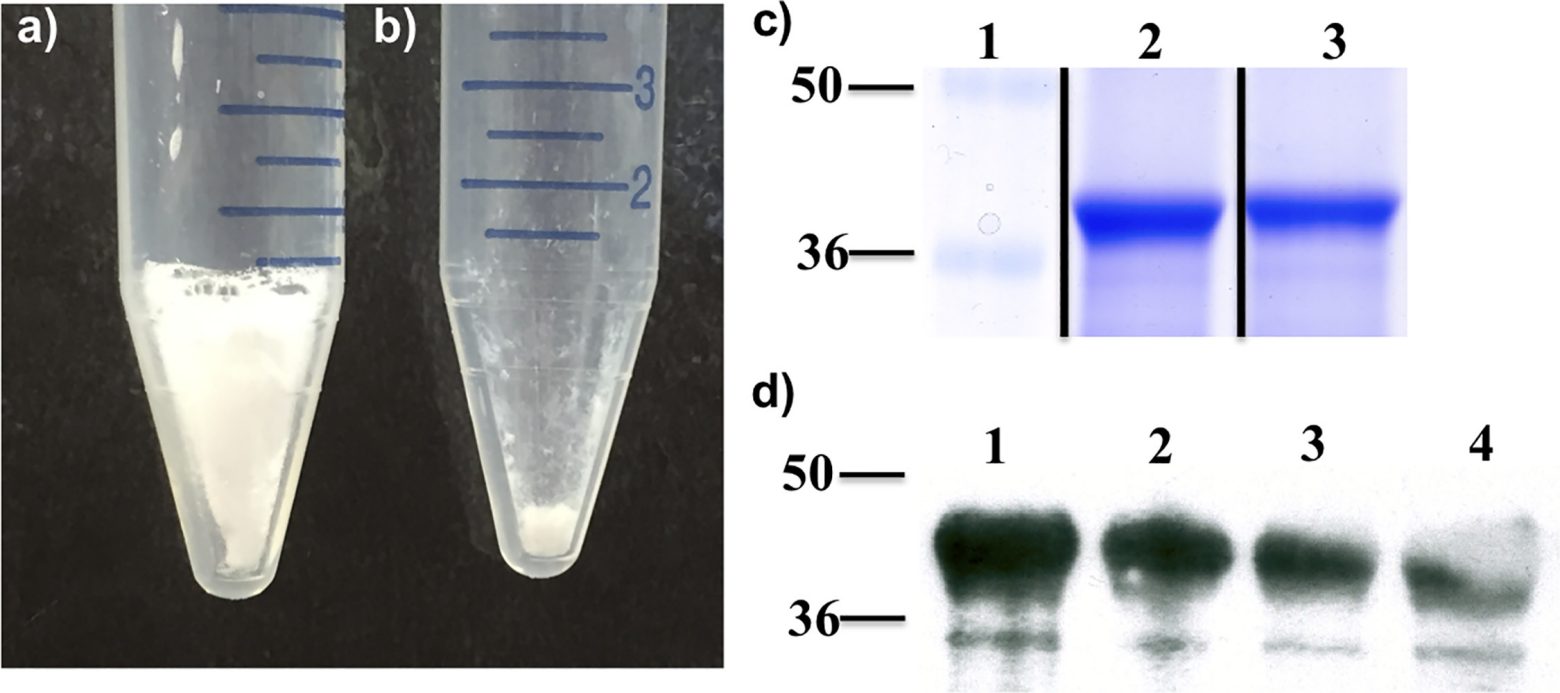
Photograph of FD oE2/SV-140 a) with 5% trehalose and 0.1% glycine; b) without excipients; c) SDS PAGE—adsorption of oE2 on SV-140, lane 1 –marker, lane 2 –oE2/SV-140 pellet, lane 3 –FD oE2/SV-140 pellet; d) Western hybridisation analysis of oE2 in the vaccine formulations, lane 1 –oE2 protein, lane 2 –oE2 plus Quil-A, lane 3 –oE2/SV-140, lane 4 –FD oE2/SV-140.

The SV-140 particles investigated in this study have been characterised previously [14,17] and they have shown to adsorb ~250 mg of oE2 protein to per g of the SV-140 vesicles. [14] Different concentrations of 5%, 10% and 20% (w/v) trehalose in combination with 0.1%, 0.5% or 1% (w/v) glycine as excipients were tested to develop FD oE2/SV-140 nanovaccine following adsorption. The samples freeze-dried with different concentrations of trehalose and glycine looked voluminous and snow-like (Fig 1A), as opposed to samples freeze-dried without excipients, which failed to form a freeze-dried cake (Fig 1B) The FD oE2/SV-140 developed with combination of 5% trehalose and 0.1% glycine as excipients reconstituted within 10 seconds on addition of 1 mL of water. However, the samples containing 10% and 20% trehalose required additional measure like shaking to obtain complete resuspension.

The SDS-PAGE analyses on the reconstituted sample freeze-dried with 5% trehalose and 0.1% glycine showed that the integrity of oE2 protein was preserved post freeze-drying (Fig 1C, lane 4). Furthermore, Western blot recognised the oE2 protein in the non-FD and the FD vaccine formulations (Fig 1D, lane 3 and 4).

**Fig 2:**
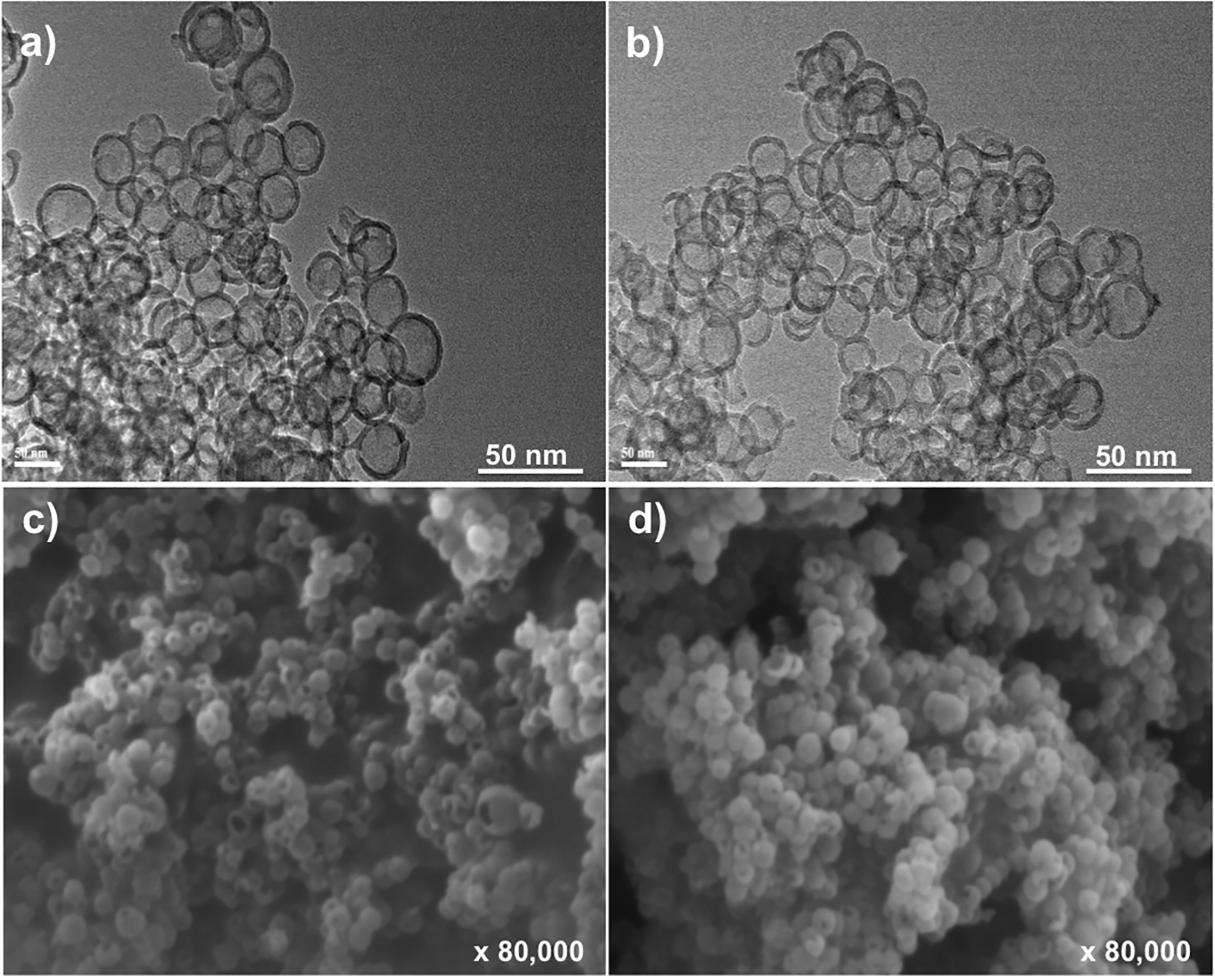
The morphology of FD SV-140 vesicles visualized by transmission electron microscope (TEM), (a) SV-140 and (b) oE2/SV-140 after lyophilisation in the presence of 5% trehalose and 0.1% glycine. The appearance of FD SV-140 by scanning electron microscope (SEM), (c) SV-140 (d) oE2/SV-140 after lyophilisation in the presence of 5% trehalose and 0.1% glycine.

The integrity of the vesicles following freeze-drying with 5% trehalose and 0.1% glycine was further confirmed by TEM and SEM analyses. The TEM results show that the silica vesicles remained intact and maintained their characteristic round shape and size of ~50 nm post freeze-drying. The SEM data indicated that the FD SV-140 and FD oE2/SV-140 samples did not suffer structural collapse (Fig 2C and 2D). The *in vivo* efficacy of the oE2/SV140 freeze-dried with 5% trehalose and 0.1% glycine and the oE2/SV-140 was investigated in a mice trial.

### Generation of antibody and cell-mediated immune responses three weeks post immunisation

**Fig 3:**
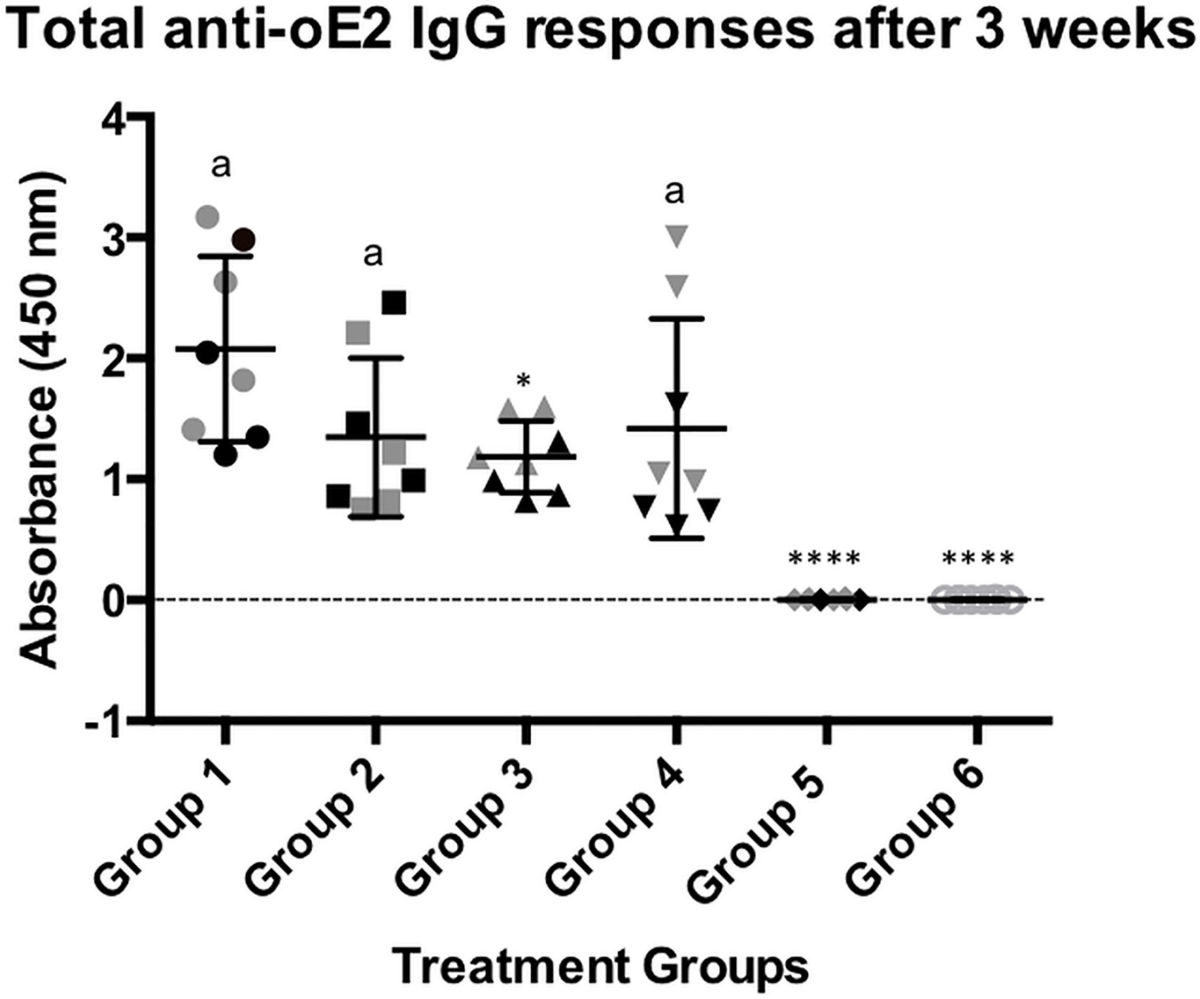
oE2-specific ELISA antibody responses in 8 mice after two subcutaneous immunisations. The individual response for each mouse is shown using a sera dilution of 1:1600. Group 1 (mouse 1 to 8) received 100 μg oE2 plus 10 μg Quil-A; Group 2 (mouse 1 to 8) received the 100 μg FD oE2 plus 10 μg Quil-A, Group 3 (mouse 1 to 8) received the oE2/SV-140 nanovaccine (100 μg oE2 adsorbed to 500 μg SV-140), Group 4 (mouse 1 to 8) received the FD oE2/SV-140 nanovaccine (100 μg oE2 adsorbed to 500 μg SV-140), Group 5 (mouse 1 to 8) received the 500 μg FD SV-140 only, Group 6 (mouse 1 to 8) was the unimmunised group and did not receive any vaccination. The black symbols in each group represent the 4 mice that were monitored for six months. The letter ‘a’ denotes that the groups were not significantly different. Groups that do not share a common letter were significantly different (* low and **** high) (*p<*0.001, unpaired t-test analysis).

To evaluate the efficacy of the oE2/SV-140 and FD oE2/SV-140 nanovaccine formulations, animals were immunised with two subcutaneous vaccinations at a three-week interval. The mice trial comprised of 48 animals divided into six groups and were immunised as described in Table 1. PI sera samples were collected prior to immunisation. Three weeks after the second vaccination sera samples were collected from all eight mice in each group and four randomly selected mice from each group were sacrificed for the analyses of IFN-γ response. The total IgG responses of the immunised mice were analysed by anti-oE2-specific ELISA assays. The animals in all the treatment groups remained healthy and in the normal weight range throughout the trial period. The non-FD and FD oE2 plus Quil-A and the FD oE2/SV-140 showed a similar trend of reduction in the level of the antibody responses and were not found to be significantly different at 1:1600 dilution (Fig 3). The FD oE2/SV-140 induced strong responses (average OD value of 1.42). Less animal-to-animal variation was observed in the mice treated with the oE2/SV-140 nanovaccine (average OD value of 1.18) (Fig 3). The average OD values for oE2 plus Quil-A and FD oE2 plus Quil-A were 2.07 and 1.34 respectively. The mice receiving the FD SV-140 only and the unimmunised group showed no oE2 specific antibody responses.

**Fig 4:**
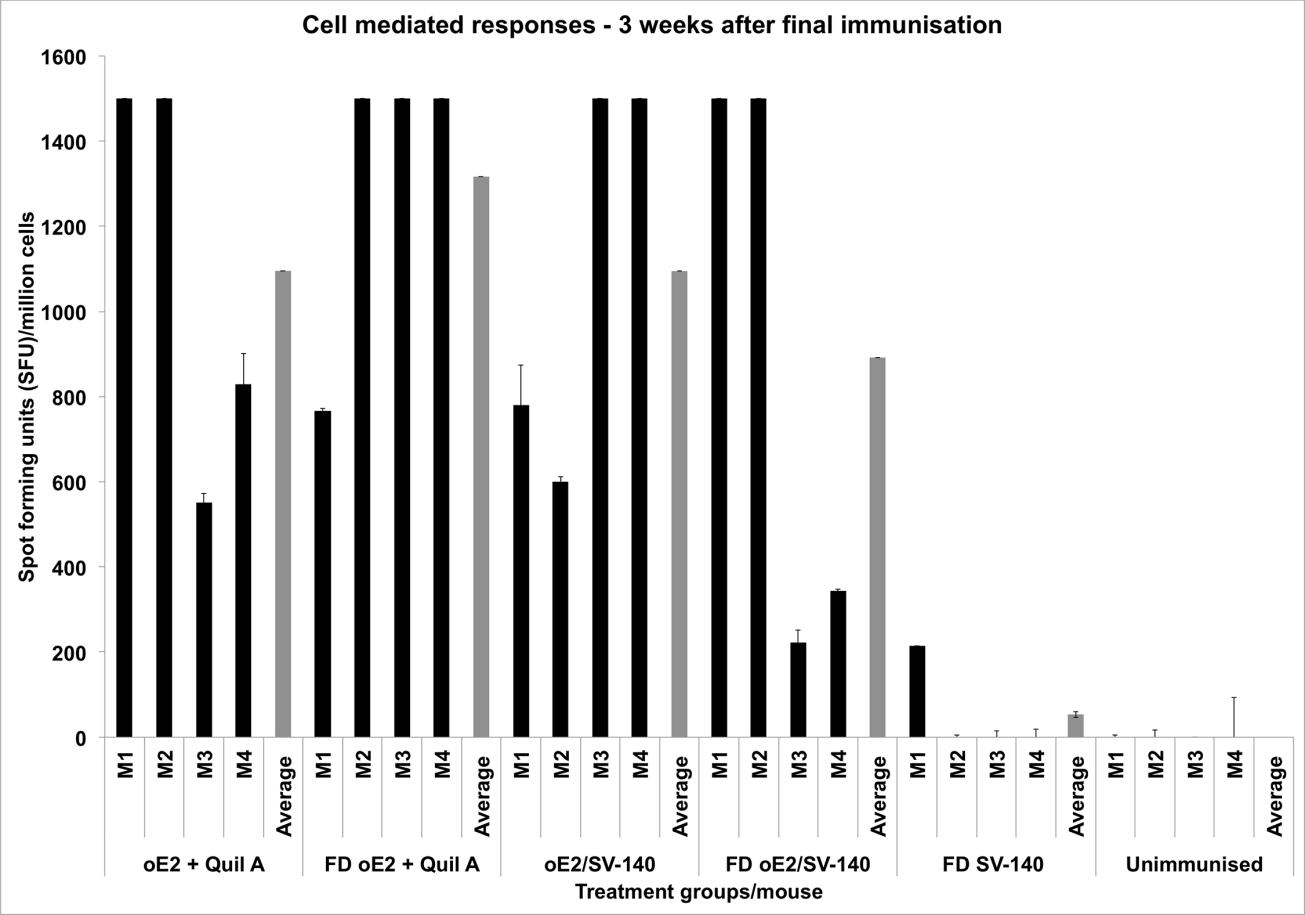
Detection of antigen specific IFN-γ secretion by ELISPOT assay of murine splenocytes from immunised mice. The black bars represent the number of cells producing IFN-γ in response to the oE2 antigen 3 weeks after the final immunisation. The grey bars show the average for each group and M1 to M4 represent the four individual mice in each group.

ELISPOT assays were used to determine the Th1 cell-mediated IFN-γ responses. Three weeks post the final immunisation spleens were collected from the four mice of the eight mice (mice shown as grey in Fig 3). The remaining four mice were retained for investigating the long-term immune responses. The number of cells producing Spot Forming Units (SFU) indicates cell-mediated immune responses to oE2 antigen. The four individual mice in oE2 plus Quil-A (551–1500 SFU/million cells) and the FD oE2 plus Quil-A (766–1500 SFU/million cells) induced strong cell-mediated responses. The oE2 specific memory responses generated by oE2/SV-140 (599–1500 SFU/million cells; average of four mice 1095 SFU/million cells) were similar to oE2 plus Quil-A (average of four mice 1094 SFU/million cells). The FD oE2/SV-140 elicited responses in the range of 222–1500 SFU/million cells; the difference in the Th1 response might be attributed to the mice-to-mice variation with 1–2 mice in each group showing a low response (Fig 4). As expected, the negative controls SV-140 freeze-dried with 5% trehalose and 0.1% glycine and unimmunised treatment groups did not generate oE2 specific responses (Fig 4).

### Generation of long-term antibody and cell-mediated immune responses six months post immunisation

**Fig 5:**
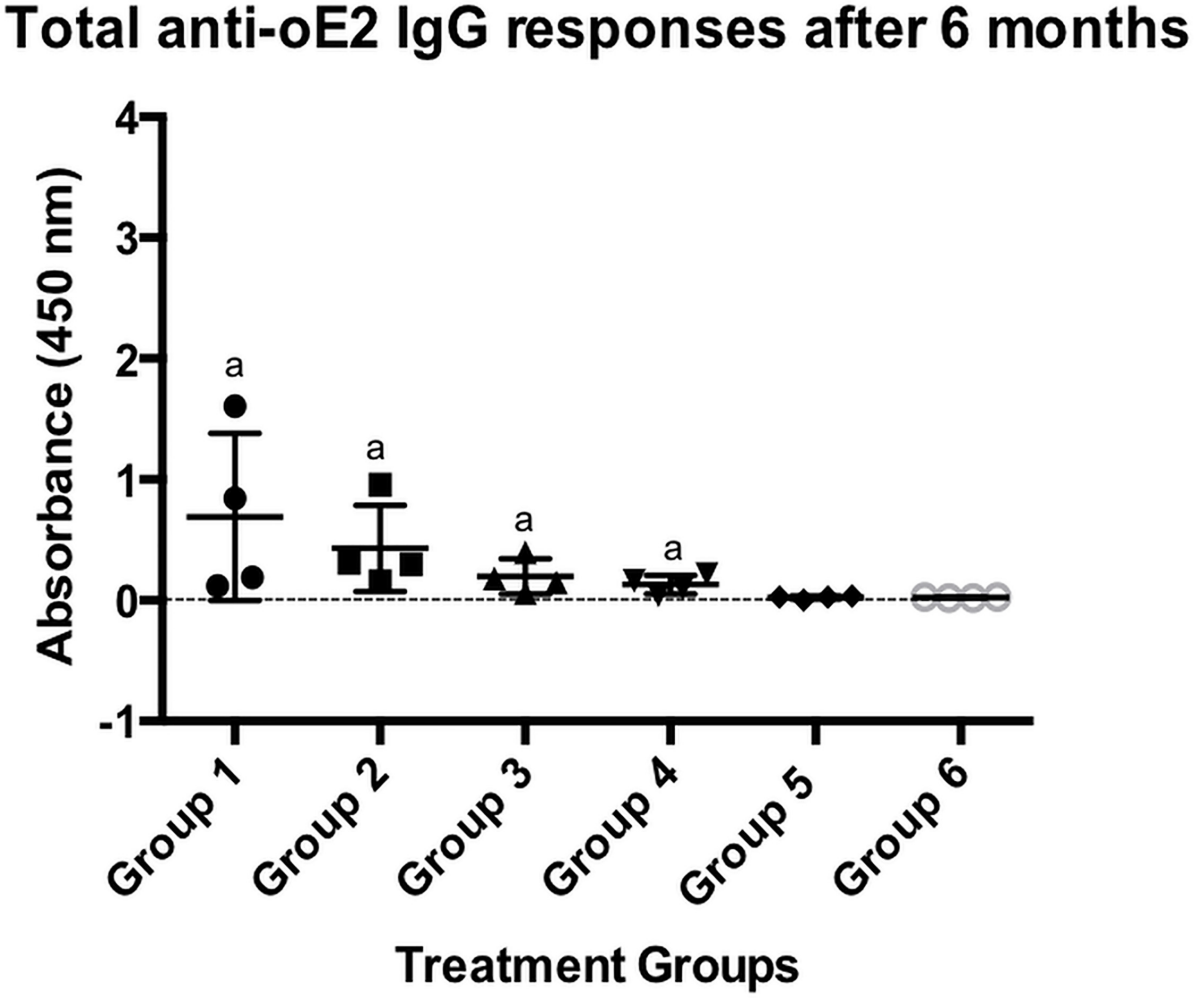
oE2-specific ELISA antibody responses in mice after two subcutaneous immunisations. The individual response for each mouse is shown using a sera dilution of 1:1600. Group 1 (mouse 5 to 8) received 100 μg oE2 plus 10 μg Quil-A; Group 2 (mouse 5 to 8) received the FD 100 μg oE2 plus 10 μg Quil-A, Group 3 (mouse 5 to 8) received the oE2 nanovaccine (100 μg oE2 adsorbed to 500 μg SV-140), Group 4 (mouse 5 to 8) received the FD oE2 nanovaccine (100 μg oE2 adsorbed to 500 μg SV-140), Group 5 (mouse 5 to 8) received the FD 500 μg SV-140, Group 6 (mouse 5 to 8) was the unimmunised group and did not receive any vaccination. The letter ‘a’ denotes that the groups were not significantly different. Groups that do not share a common letter were significantly different (*p<*0.001, unpaired t-test analysis).

To monitor the long-term responses sera samples were collected once every four weeks for up to six months after the final second immunisation. oE2-specific humoral immune responses were measured by ELISA at the different time points. As expected a gradual trend of reduction in the antibody responses generated by the oE2 injected with conventional adjuvant Quil-A as well as SV-140 was observed at 7 weeks, 11 weeks, 15 weeks and 19 weeks (S1 Fig). Antibody responses to the oE2 antigen generated by the oE2 plus Quil-A, FD oE2 plus Quil-A, oE2/SV-140 as well as FD oE2/SV-140 reduced significantly by the end of six months, however, detectable level of antibody responses were still generated at 1:1600 dilution by both oE2/SV-140 (average OD value of 0.19) and FD oE2/SV-140 (average OD value of 0.13, Fig 5) at the six-month time point. The oE2 plus Quil-A induced strong antibody responses (average OD value of 0.68), which could be due to the two mice in this group showing higher response. The FD oE2 plus Quil-A also showed high antibody responses (average OD values of 0.43). The FD SV-140 alone and the unimmunised treatments did not generate oE2 specific antibody responses. This is an important finding as for the first time we have demonstrated that the oE2 adsorbed SV-140 induced long-term antibody responses in mice and also that freeze-drying the oE2/SV-140 nanovaccine with 5% trehalose and 0.1% glycine maintained the immunological integrity of oE2 protein.

**Fig 6:**
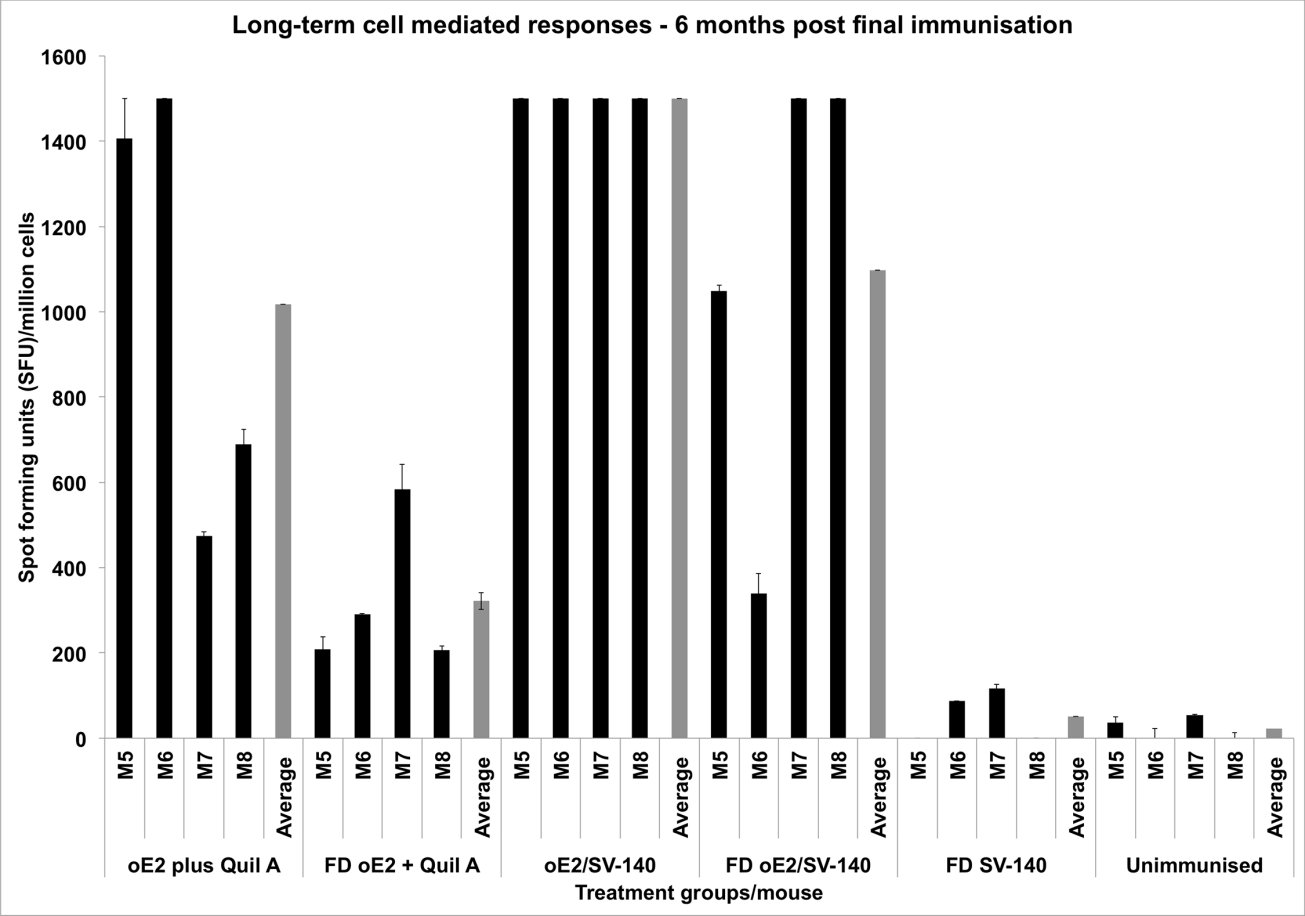
Detection of antigen specific IFN-γ secretion by ELISPOT assay of murine splenocytes from immunised mice. The bars represent the number of cells producing IFN-γ in response to the oE2 antigen six months after the final immunisation. The grey bars show the average for each group and M5 to M8 represent the four individual mice in each group.

Generation of long-term cell-mediated immune response is crucial as it shows uptake of the antigen by the antigen presenting cells, which is an essential process for developing immunity to invading pathogens. To determine the long term cell-mediated immune responses spleens were collected from the four sacrificed mice at the end of the trial at six months following the second immunisation. The negative controls FD SV-140 alone and unimmunised did not generate oE2 specific cell-mediated responses (Fig 6). The oE2/SV-140 generated very strong long-term Th1 responses, the average value was found to be significantly higher (1500 SFU/million cells) than all the treatment groups. The FD oE2/SV-140 treatment group showed some variation in Th1 cell-mediated immune responses to oE2 antigen as two mice generated low level responses of 340–1049 SFU/million cells while the other two mice generated high level responses of 1500 SFU/million cells. Similarly, two mice in the oE2 plus Quil-A group induced low level of responses (473–690 SFU/million cells) whereas the other two mice generated high level of responses (1406–1500 SFU/million cells), this varied response can be attributed to mouse-to-mouse variation. All the four mice in the FD oE2 plus Quil-A group induced low level responses of 206–583 SFU/million cells.

Immunohistochemistry analyses of mice spleen sections were conducted to determine semi quantitative IgG responses and the representative results are shown in S2 Fig. The IgG responses appeared stronger at both the time points (three weeks and six months) with oE2/SV-140 (S2E and S2F Fig) compared to the positive control oE2 plus Quil-A (S2A and S2B Fig). In addition, the histopathology results demonstrated that administration of the oE2/SV-140, FD oE2/SV-140 and FD SV-140 alone did not have a detrimental effect on the mouse organs at three weeks as well as six months as the sections of mice injected with the nanoformulations looked similar to the unimmunised treatment group (S3A and S3B Fig).

The effectiveness of the non-FD and the FD SV-140 as self-adjuvants and efficient antigen delivery vehicles were demonstrated by the ELISA and ELISpot assay results which showed good oE2-specific antibody and cell-mediated immune responses at three week and six month time points. This is a significant finding as for the first time we have shown that the SV can induce long-term humoral and cell-mediated immune responses.

## Discussion

For subunit vaccines to be successful commercially it is important that they induce humoral and cell-mediated responses as well as sustain long-term immunogenicity. This research work aims on developing a freeze-dried and non freeze-dried veterinary subunit vaccine delivery system, using SV-140 as adjuvants and delivery vehicles for BVDV E2 antigen, with the potential to initiate long-term immunity. Here, for the first time we have shown that the oE2/SV-140 formulation induced humoral and cell-mediated immune responses for up to six months in mice after two subcutaneous immunisations. In addition, FD of the oE2/SV-140 formulation also generated long-term antibody and cell-mediated immune responses for up to six months.

In a recent study, we reported the capacity of SV-140 as nanocarriers for efficient adsorption of oE2; SV-140 materials displayed excellent cellular uptake proficiency and were found to be non-toxic on MDBK cells. The oE2/SV-140 formulation was then tested in mice and the animals were vaccinated with three vaccinations at two week intervals subcutaneously with 50 μg oE2/250 μg SV-140 and 50 μg oE2 plus 10 μg Quil-A as positive control. The oE2/SV-140 induced higher anti-oE2 IgG as well as IFN-γ responses compared to traditional adjuvant Quil-A, demonstrating the potential of SV-140 as both efficient vaccine delivery vehicles and potent adjuvants. [14] These results encouraged us to test the long-term efficacy of the oE2/SV-140 nanovaccine, as generation of long-term immunity is a requisite for the development of a successful subunit vaccine. We also developed and tested the ability of the FD oE2/SV-140 nanovaccine to generate immunity in mice to address the issue of cold chain storage of subunit vaccines.

Freeze-drying is considered an excellent good technique to improve the long-term stability of the nanoformulations but it is a very complex process. Post freeze-drying the integrity of the nanoparticles and protein needs to be investigated. Various studies have reported the use of trehalose in combination to preserve the immunogenicity of proteins such as lactose dehydrogenase, hepatitis B surface antigen (HBsAg) and human serum albumin. [19,20,21] Likewise, glycine also has been used successfully for freeze-drying of model proteins lactate dehydrogenase and glucose 6-phosphate dehydrogenase in a sucrose-glycine based excipient system. [22] In the past, we have observed that 5% trehalose along with either 1% PEG8000 helped preserve silica nanoparticles and immunogenicity of OVA protein. [13] For freeze-drying, oE2 adsorbed SV-140 the combination of 5% trehalose and 0.1% glycine was found to be suitable as it preserved the structural integrity of the vesicles as well as the integrity of the oE2 protein. As determined by western analyses the antigenicity of the FD BVDV oE2 adsorbed on SV-140 was preserved (Fig 1D lane 4). Sameti *et al*. demonstrated that the activity of cationic silica nanoparticles was preserved when freeze-dried with either trehalose or glycerol. [23] The freeze-drying process did not have an adverse affect on the ability of the dried formulations the oE2/SV-140 and SV-140 to spontaneously go into the solution upon hydration. Furthermore, as observed by TEM and SEM the structural integrity of the SV-140 adsorbed with oE2 and SV-140 before and after freeze-drying was well preserved. In addition, previous work from our laboratory has demonstrated that the FD silica nanovaccine formulations (OVA/AM-41 and oE2/HMSA) induced both antibody and cell-mediated immune responses in mice [13] and sheep (personal communication).

In the current study, mice were administered with two vaccinations of the non-FD and the FD 100 μg oE2/500 μg SV-140 formulations at three week intervals. The animals were maintained for up to six months post the final immunisation and mice in all the groups remaining healthy throughout the trial period. Quantitative toxicity analyses on SV-140 nanovaccine formulations conducted at 0.02 mg/mL and 0.01 mg/mL showed that >85% of the MDBK cells remained viable. [14] Both oE2/SV-140 and the FD oE2/SV-140 induced oE2 specific antibody responses (1:1600) at three weeks (average OD range of 1.18 vs. 1.42) and six months (average OD range of 0.19 vs. 0.13) after the final second immunisation (Figs 3 and 5). As expected, with time the oE2/SV-140, oE2 plus Quil-A, the FD oE2/SV-140 and FD oE2 plus Quil-A showed a gradual trend of reduction in the antibody response. Hollow mesoporous silica nanoparticles used to deliver Porcine Circovirus Type 2 ORF2 protein in mice showed significant reduction in the antibody titres at the six week time point post immunisation. [24] The cell-mediated response, which is very important part of the anti-viral response, was found to be strong with oE2/SV-140 and FD oE2/SV-140 at the three week as well as six month time points (222–1500 SFU/million cells) (Figs 4 and 6). The uniformly strong high cell-mediated response induced by the four mice (1500 SFU/million cells) in the oE2/SV-140 could be due to the sustained release of the antigen from the vesicles. The density and intensity of the signal could not be directly quantified by the ELISPOT reader. In order to include these animals in the subsequent analyses each was assigned the arbitrary value of 1500 SFU/million cells based on the upper detection level of the ELISPOT reader. These assigned values were within one standard deviation of the average values of the ELISPOT controls stimulated with ConA and were therefore considered to be realistic estimations. Even though, the oE2 specific cell-mediated immune responses with the oE2/SV-140 were higher than the FD oE2/SV-140 at 3 weeks as well as six months, this study confirms the ability of FD oE2/SV-140 to induce long-term humoral and cell-mediated immune responses. Tonnis *et al*. [19] demonstrated similar finding as they found that the freeze-dried aluminum hydroxide adjuvanted HBsAg formulation did not induce high immune responses but was able to induce both Th1 and Th2 responses. [19]

The elicitation of total IgG response was further confirmed by fluorescent FITC staining of the spleen sections, which showed that both oE2/SV-140 as well as FD oE2/SV-140 generated strong antibody responses at three week and six month time points (S2 Fig). Previously, we have shown that administration of 150 μg AM-41 silica nanoparticles did not cause any morphological changes in the mice organs. [16] Similarly, the histopathology studies on different organs of mice immunised with the oE2/SV-140, FD oE2/SV-140 and FD SV-140 nanovaccine treatments groups confirmed that the 50 nm SV are biocompatible materials and that administration of 500 μg SV-140 vesicles did not have deleterious side effects (S3 Fig).

In conclusion, the elicitation of humoral and cell-mediated responses by the non-FD and FD oE2 adsorbed to SV-140 for up to six months after the final second vaccination, further proves the potential of silica vesicles as a promising new generation adjuvant and delivery vehicle for the development of BVDV subunit vaccine. The efficacy of this adjuvant platform suggests that it can be applied to produce cost effective veterinary subunit vaccines with improved shelf life.

## Supporting Information

S1 Fig oE2-specific ELISA antibody responses in mice after two subcutaneous immunisations. The individual response for each mouse is shown using a sera dilution of 1:1600. Group 1 (mouse 5 to 8) received 100 μg oE2 plus 10 μg Quil-A; Group 2 (mouse 5 to 8) received the FD 100 μg oE2 plus 10 μg Quil-A, Group 3 (mouse 5 to 8) received the oE2 nanovaccine (100 μg oE2 adsorbed to 500 μg SV-140), Group 4 (mouse 5 to 8) received the FD oE2 nanovaccine (100 μg oE2 adsorbed to 500 μg SV-140), Group 5 (mouse 5 to 8) received the FD 500 μg SV-140, Group 6 (mouse 5 to 8) was the unimmunised group and did not receive any vaccination.
(PDF)

S2 Fig Representative immunohistochemistry analyses to determine the induction of total IgG in the spleen sections of the vaccinated animals after three weeks and six months post the final immunisation, oE2 plus Quil A (a) and (b); FD oE2 plus Quil A (c) and (d); oE2/SV-140 (e) and (f); FD oE2/SV-140 (g) and (h); FD SV-140 (i) and (j); unimmnised (k) and (l). (PDF)

S3 Fig Histopathology studies of tissue organs from a mouse injected with nanovaccine immunisations; A) Three weeks post the final immunisation, organs fixed in formalin were harvested from two mice for each treatment group and embedded in paraffin, sections were stained with hematoxylin and eosin stain. i) Heart, ii) Injection sites, iii) Kidney, iv) Liver. B) Six months post the final immunisation, organs fixed in formalin were harvested from two mice for each treatment group and embedded in paraffin, sections were stained with hematoxylin and eosin stain. i) Heart, ii) Injection sites, iii) Kidney, iv) Liver. (PDF)

S4 Fig End point titer data of terminal sera bleeds. All the mice were administered 100 μL of two vaccine doses at 3 week intervals at the tail base. Group 1 (mouse 1 to 8) received 100 μg oE2 plus 10 μg Quil-A; Group 2 (mouse 1 to 8) received the FD 100 μg oE2 plus 10 μg Quil-A, Group 3 (mouse 1 to 8) received the oE2 nanovaccine (100 μg oE2 adsorbed to 500 μg SV-140), Group 4 (mouse 1 to 8) received the FD oE2 nanovaccine (100 μg oE2 adsorbed to 500 μg SV-140), Group 5 (mouse 1 to 8) received the FD 500 μg SV-140, Group 6 (mouse 1 to 8) was the unimmunised group and did not receive any vaccination. Sera of individual animals were diluted from 1:100 to 1:6400.
(PDF)

S5 Fig Full gel image of Fig 1C and 1D. (PDF)

S1 Table Different concentrations of Trehalose and Glycine tested to freeze-dry oE2/SV-140. (PDF)
