# Correction: Silica Vesicle Nanovaccine Formulations Stimulate Long-Term Immune Responses to the *Bovine Viral Diarrhoea* Virus E2 Protein

**DOI:** 10.1371/journal.pone.0146631

**Published:** 2016-01-05

**Authors:** Karishma T. Mody, Donna Mahony, Antonino S. Cavallaro, Jun Zhang, Bing Zhang, Timothy J. Mahony, Chengzhong Yu, Neena Mitter

The images for S2, S3 and S4 Figs are incorrect. Please view the correct S2, S3 and S4 Figs here.

## Supporting Information

S2 Fig Representative immunohistochemistry analyses to determine the induction of total IgG in the spleen sections of the vaccinated animals after three weeks and six months post the final immunisation, oE2 plus Quil A (a) and (b); FD oE2 plus Quil A (c) and (d); oE2/SV-140 (e) and (f); FD oE2/SV-140 (g) and (h); FD SV-140 (i) and (j); unimmnised (k) and (l). (PDF)

S3 Fig Histopathology studies of tissue organs from a mouse injected with nanovaccine immunisations; A) Three weeks post the final immunisation, organs fixed in formalin were harvested from two mice for each treatment group and embedded in paraffin, sections were stained with hematoxylin and eosin stain. i) Heart, ii) Injection sites, iii) Kidney, iv) Liver. B) Six months post the final immunisation, organs fixed in formalin were harvested from two mice for each treatment group and embedded in paraffin, sections were stained with hematoxylin and eosin stain. i) Heart, ii) Injection sites, iii) Kidney, iv) Liver.
(PDF)

S4 Fig End point titer data of terminal sera bleeds. All the mice were administered 100 μL of two vaccine doses at 3 week intervals at the tail base. Group 1 (mouse 1 to 8) received 100 μg oE2 plus 10 μg Quil-A; Group 2 (mouse 1 to 8) received the FD 100 μg oE2 plus 10 μg Quil-A, Group 3 (mouse 1 to 8) received the oE2 nanovaccine (100 μg oE2 adsorbed to 500 μg SV-140), Group 4 (mouse 1 to 8) received the FD oE2 nanovaccine (100 μg oE2 adsorbed to 500 μg SV-140), Group 5 (mouse 1 to 8) received the FD 500 μg SV-140, Group 6 (mouse 1 to 8) was the unimmunised group and did not receive any vaccination. Sera of individual animals were diluted from 1:100 to 1:6400.
(PDF)
