# Supplementary material for: Silica Vesicle Nanovaccine Formulations Stimulate Long-Term Immune Responses to the Bovine Viral Diarrhoea Virus E2 Protein
Source: PLoS One. 2015 Dec 2;10(12):e0143507. doi: 10.1371/journal.pone.0143507 (PMC4668082; doi:10.1371/journal.pone.0143507)
Supplement: S1 Fig — The individual response for each mouse is shown using a sera dilution of 1:1600. Group 1 (mouse 5 to 8) received 100 μg oE2 plus 10 μg Quil-A; Group 2 (mouse 5 to 8) received the FD 100 μg oE2 plus 10 μg Quil-A, Group 3 (mouse 5 to 8) received the oE2 nanovaccine (100 μg oE2 adsorbed to 500 μg SV-140), Group 4 (mouse 5 to 8) received the FD oE2 nanovaccine (100 μg oE2 adsorbed to 500 μg SV-140), Group 5 (mouse 5 to 8) received the FD 500 μg SV-140, Group 6 (mouse 5 to 8) was the unimmunised group and did not receive any vaccination. (PDF) [file pone.0143507.s001.pdf]

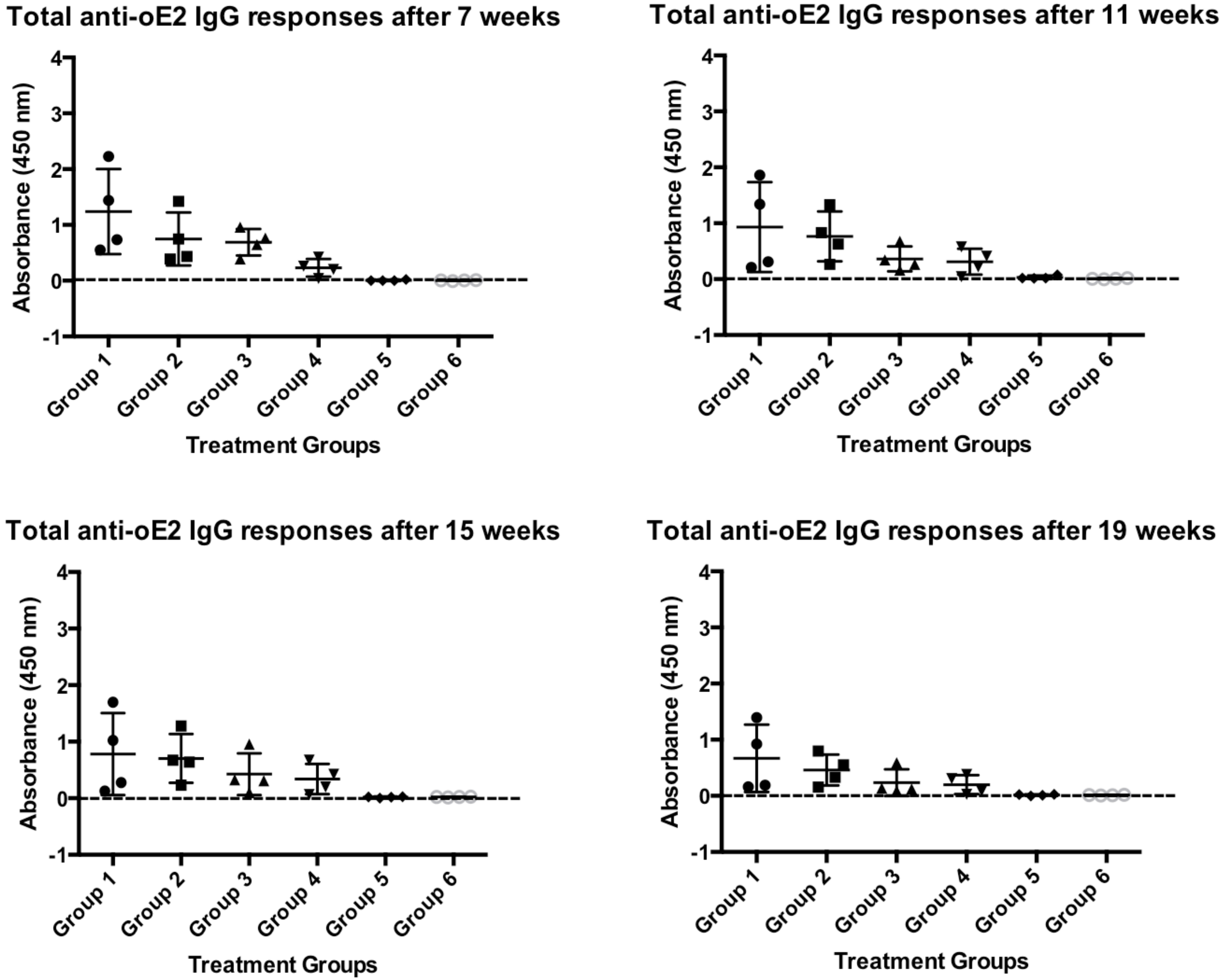

S1 Fig. oE2-specific ELISA antibody responses in mice after two subcutaneous immunisations. The individual response for each mouse is shown using a sera dilution of 1:1600. Group 1 (mouse 5 to 8) received 100 µg oE2 plus 10 µg Quil-A; Group 2 (mouse 5 to 8) received the FD 100 µg oE2 plus 10 µg Quil-A, Group 3 (mouse 5 to 8) received the oE2 nanovaccine (100 µg oE2 adsorbed to 500 µg SV-140), Group 4 (mouse 5 to 8) received the FD oE2 nanovaccine (100 µg oE2 adsorbed to 500 µg SV-140), Group 5 (mouse 5 to 8) received the FD 500 µg SV-140, Group 6 (mouse 5 to 8) was the unimmunised group and did not receive any vaccination.
