# Supplementary material for: Silica Vesicle Nanovaccine Formulations Stimulate Long-Term Immune Responses to the Bovine Viral Diarrhoea Virus E2 Protein
Source: PLoS One. 2015 Dec 2;10(12):e0143507. doi: 10.1371/journal.pone.0143507 (PMC4668082; doi:10.1371/journal.pone.0143507)
Supplement: S2 Fig — (PDF) [file pone.0143507.s002.pdf]

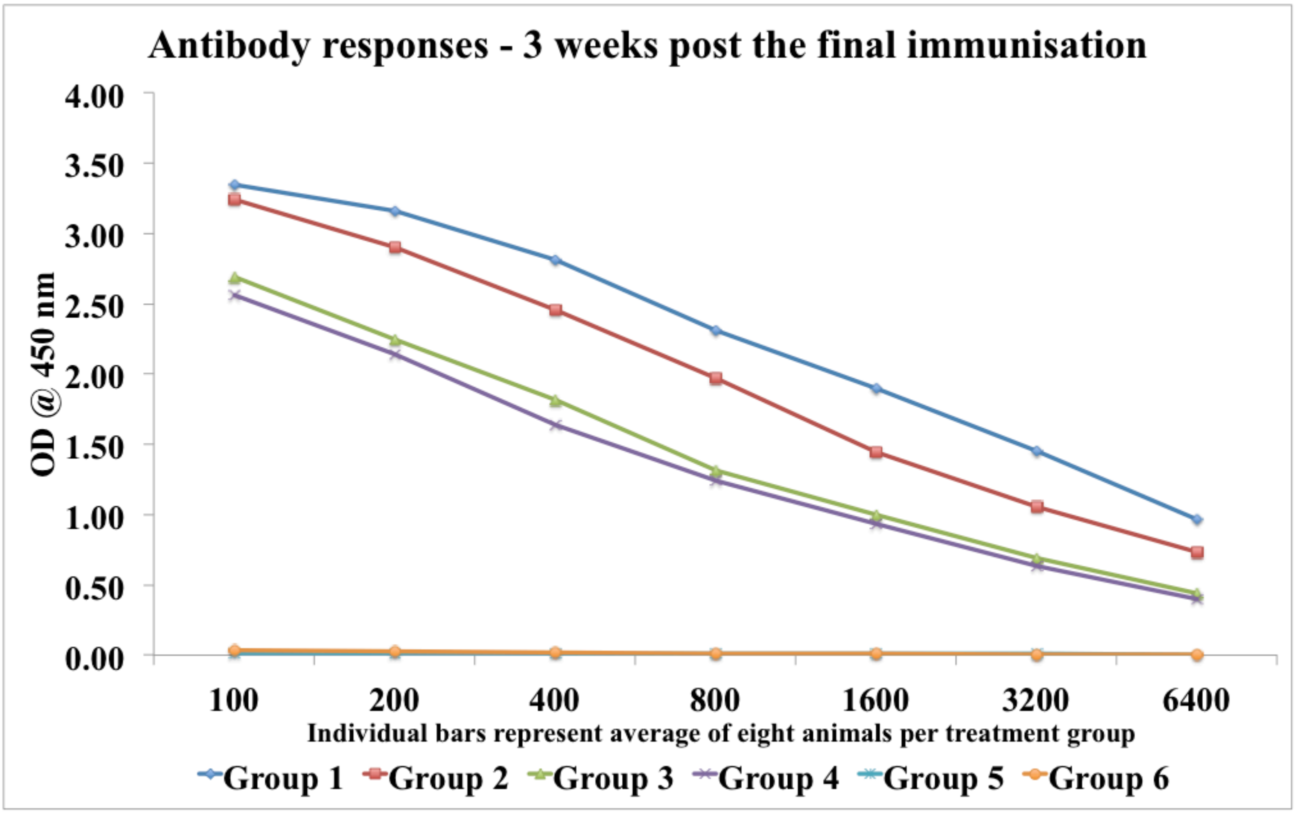
Antibody responses - 3 weeks post the final immunisation
OD @ 450 nm
4.00
3.50
3.00
2.50
2.00
1.50
1.00
0.50
0.00
100
200
400
800
1600
3200
6400
Individual bars represent average of eight animals per treatment group
Group 1
Group 2
Group 3
Group 4
Group 5
Group 6

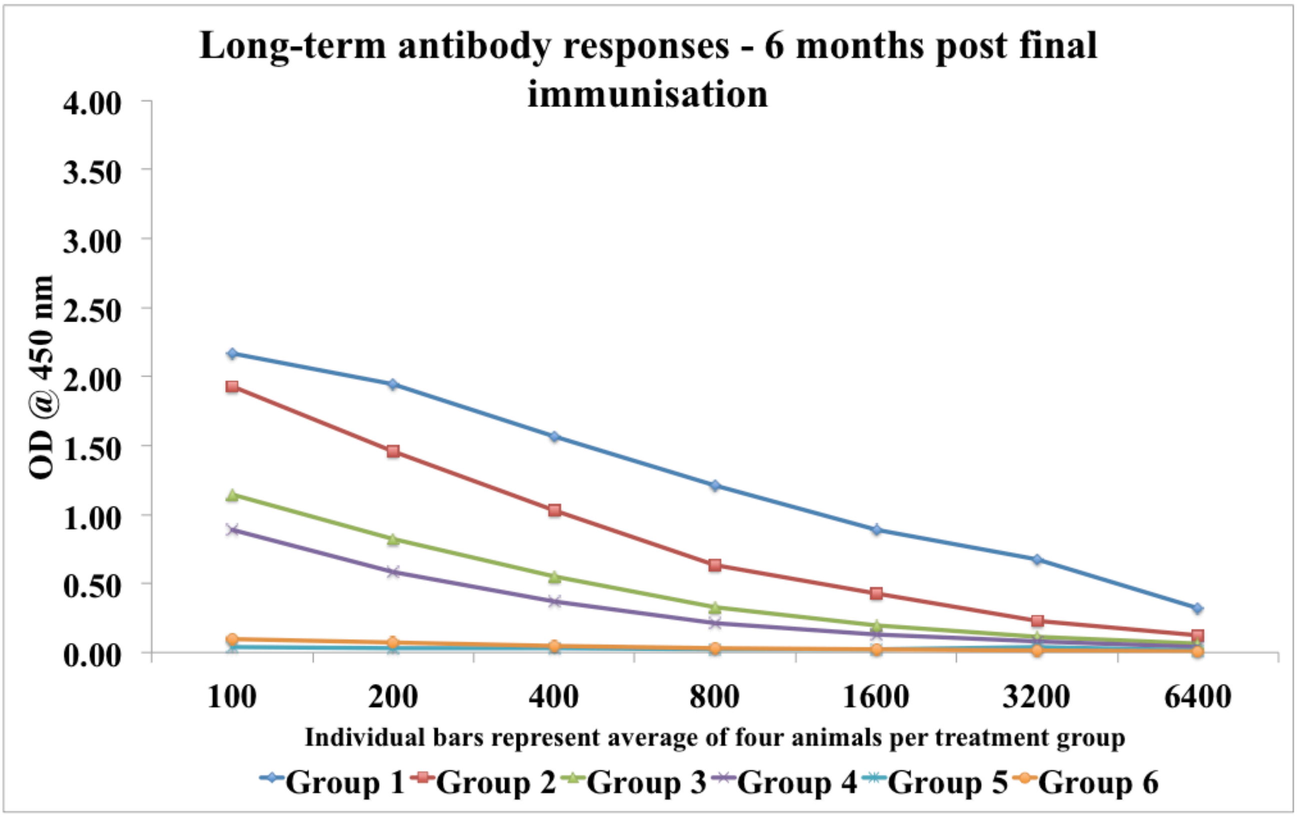
Long-term antibody responses - 6 months post final immunisation
OD @ 450 nm
4.00
3.50
3.00
2.50
2.00
1.50
1.00
0.50
0.00
100
200
400
800
1600
3200
6400
Individual bars represent average of four animals per treatment group
Group 1
Group 2
Group 3
Group 4
Group 5
Group 6
