# Supplementary figures and images for: Silica Vesicle Nanovaccine Formulations Stimulate Long-Term Immune Responses to the Bovine Viral Diarrhoea Virus E2 Protein
Source: PLoS One. 2015 Dec 2;10(12):e0143507. doi: 10.1371/journal.pone.0143507 (PMC4668082; doi:10.1371/journal.pone.0143507)

**After 3 weeks**

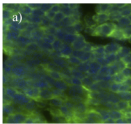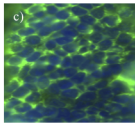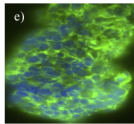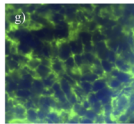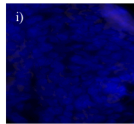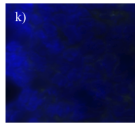

**After 6 months**

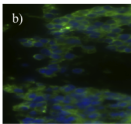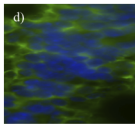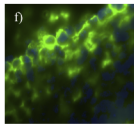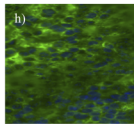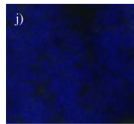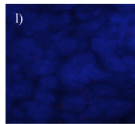

Supplement: S3 Fig — (PDF) [file pone.0143507.s003.pdf]

A)

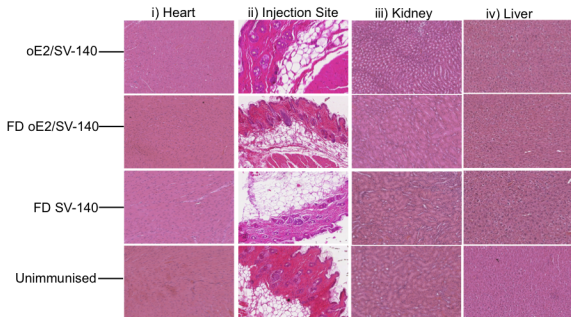

B)

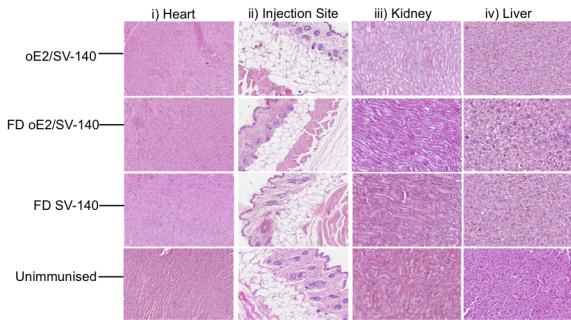

Supplement: S4 Fig — All the mice were administered 100 μL of two vaccine doses at 3 week intervals at the tail base. Group 1 (mouse 1 to 8) received 100 μg oE2 plus 10 μg Quil-A; Group 2 (mouse 1 to 8) received the FD 100 μg oE2 plus 10 μg Quil-A, Group 3 (mouse 1 to 8) received the oE2 nanovaccine (100 μg oE2 adsorbed to 500 μg SV-140), Group 4 (mouse 1 to 8) received the FD oE2 nanovaccine (100 μg oE2 adsorbed to 500 μg SV-140), Group 5 (mouse 1 to 8) received the FD 500 μg SV-140, Group 6 (mouse 1 to 8) was the unimmunised group and did not receive any vaccination. Sera of individual animals were diluted from 1:100 to 1:6400. (PDF) [file pone.0143507.s004.pdf]
