# Supplementary material for: Silica Vesicle Nanovaccine Formulations Stimulate Long-Term Immune Responses to the Bovine Viral Diarrhoea Virus E2 Protein
Source: PLoS One. 2015 Dec 2;10(12):e0143507. doi: 10.1371/journal.pone.0143507 (PMC4668082; doi:10.1371/journal.pone.0143507)
Supplement: S5 Fig — (PDF) [file pone.0143507.s005.pdf]

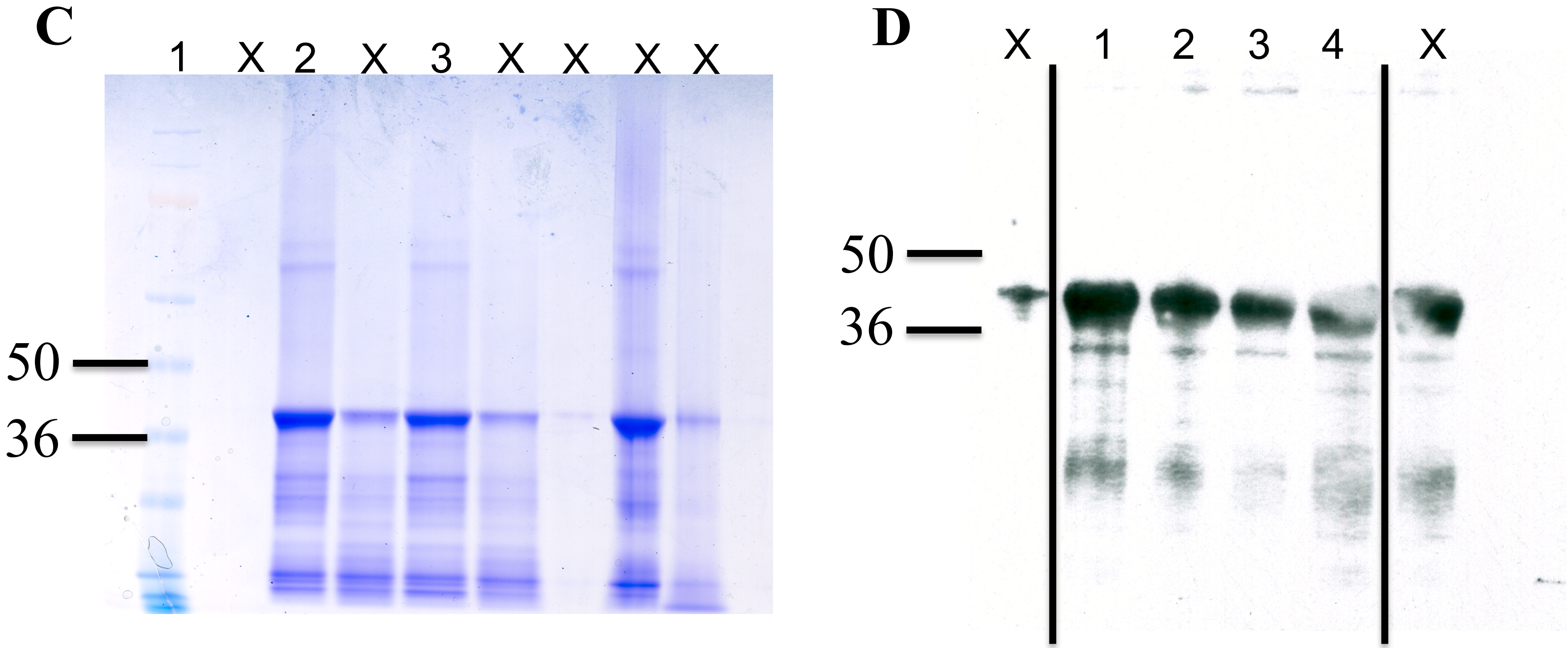

Fig 1. Photograph of FD oE2/SV-140 **a)** with 5% trehalose and 0.1% glycine; **b)** without excipients; **c)** SDS PAGE - adsorption of oE2 on SV-140, lane 1 – marker, lane 2 – oE2/SV-140 pellet, lane 3 – FD oE2/SV-140 pellet; **d)** Western hybridisation analysis of oE2 in the vaccine formulations, lane 1 – oE2 protein, lane 2 – oE2 plus Quil-A, lane 3 – oE2/SV-140, lane 4 – FD oE2/SV-140.
