# Supplementary material for: Silica Vesicle Nanovaccine Formulations Stimulate Long-Term Immune Responses to the Bovine Viral Diarrhoea Virus E2 Protein
Source: PLoS One. 2015 Dec 2;10(12):e0143507. doi: 10.1371/journal.pone.0143507 (PMC4668082; doi:10.1371/journal.pone.0143507)
Supplement: S1 Table — (PDF) [file pone.0143507.s006.pdf]

S1 Table. Different concentrations of Trehalose and Glycine tested to freeze-dry oE2/SV-140

Excipients added	0.1% Glycine	0.5% Glycine	1% Glycine
5% Trehalose	*	*	*
10% Trehalose	*	*	*
20% Trehalose	*	*	*
