# Supplementary figures and images for: Correction: Silica Vesicle Nanovaccine Formulations Stimulate Long-Term Immune Responses to the Bovine Viral Diarrhoea Virus E2 Protein
Source: PLoS One. 2016 Jan 5;11(1):e0146631. doi: 10.1371/journal.pone.0146631 (PMC4701184; doi:10.1371/journal.pone.0146631)

**After 3 weeks**

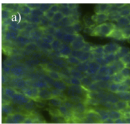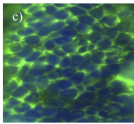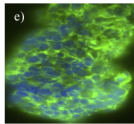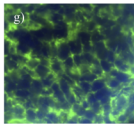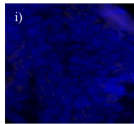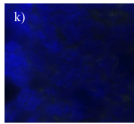

**After 6 months**

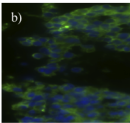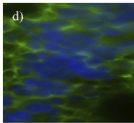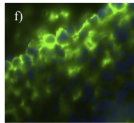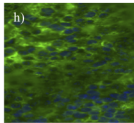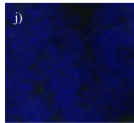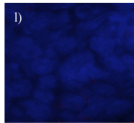

Supplement: S2 Fig — (PDF) [file pone.0146631.s001.pdf]

A)

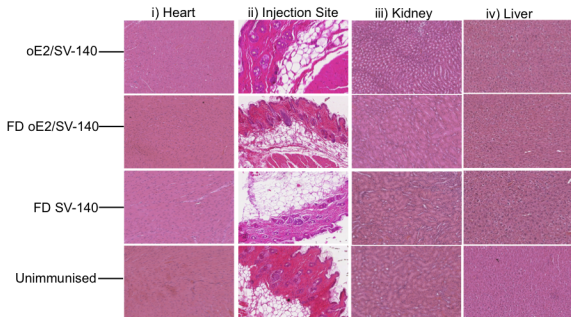

B)

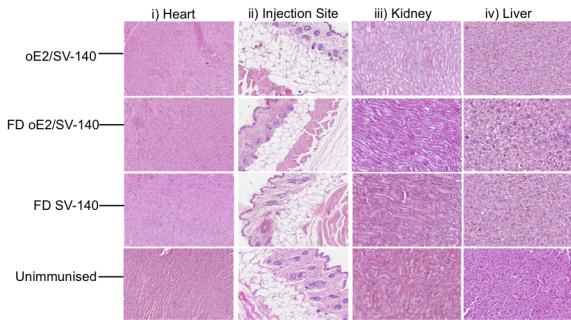

Supplement: S3 Fig — Histopathology studies of tissue organs from a mouse injected with nanovaccine immunisations; A) Three weeks post the final immunisation, organs fixed in formalin were harvested from two mice for each treatment group and embedded in paraffin, sections were stained with hematoxylin and eosin stain. i) Heart, ii) Injection sites, iii) Kidney, iv) Liver. B) Six months post the final immunisation, organs fixed in formalin were harvested from two mice for each treatment group and embedded in paraffin, sections were stained with hematoxylin and eosin stain. i) Heart, ii) Injection sites, iii) Kidney, iv) Liver. (PDF) [file pone.0146631.s002.pdf]
