# Supplementary material for: Correction: Silica Vesicle Nanovaccine Formulations Stimulate Long-Term Immune Responses to the Bovine Viral Diarrhoea Virus E2 Protein
Source: PLoS One. 2016 Jan 5;11(1):e0146631. doi: 10.1371/journal.pone.0146631 (PMC4701184; doi:10.1371/journal.pone.0146631)
Supplement: S4 Fig — All the mice were administered 100 μL of two vaccine doses at 3 week intervals at the tail base. Group 1 (mouse 1 to 8) received 100 μg oE2 plus 10 μg Quil-A; Group 2 (mouse 1 to 8) received the FD 100 μg oE2 plus 10 μg Quil-A, Group 3 (mouse 1 to 8) received the oE2 nanovaccine (100 μg oE2 adsorbed to 500 μg SV-140), Group 4 (mouse 1 to 8) received the FD oE2 nanovaccine (100 μg oE2 adsorbed to 500 μg SV-140), Group 5 (mouse 1 to 8) received the FD 500 μg SV-140, Group 6 (mouse 1 to 8) was the unimmunised group and did not receive any vaccination. Sera of individual animals were diluted from 1:100 to 1:6400. (PDF) [file pone.0146631.s003.pdf]

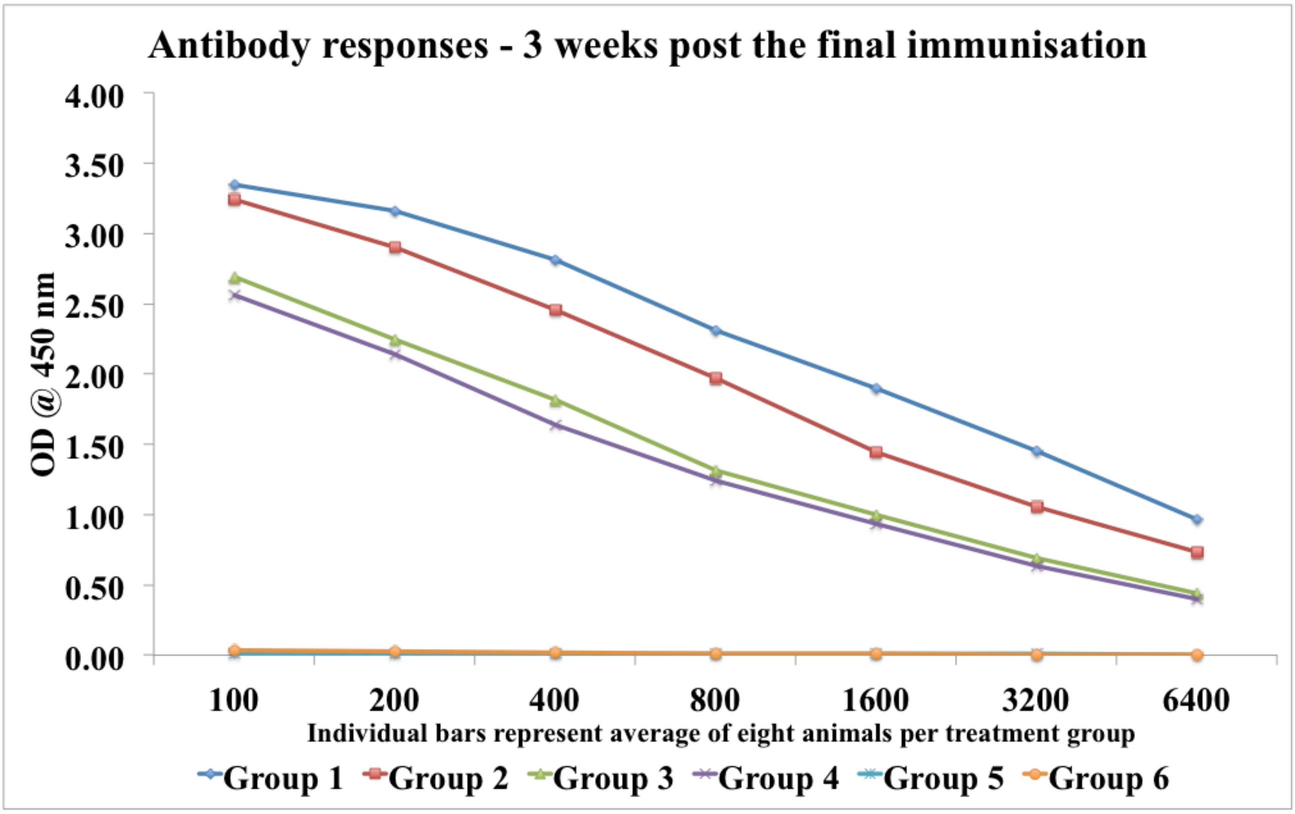

Antibody responses - 3 weeks post the final immunisation
OD @ 450 nm
4.00
3.50
3.00
2.50
2.00
1.50
1.00
0.50
0.00
100
200
400
800
1600
3200
6400
Individual bars represent average of eight animals per treatment group
Group 1
Group 2
Group 3
Group 4
Group 5
Group 6

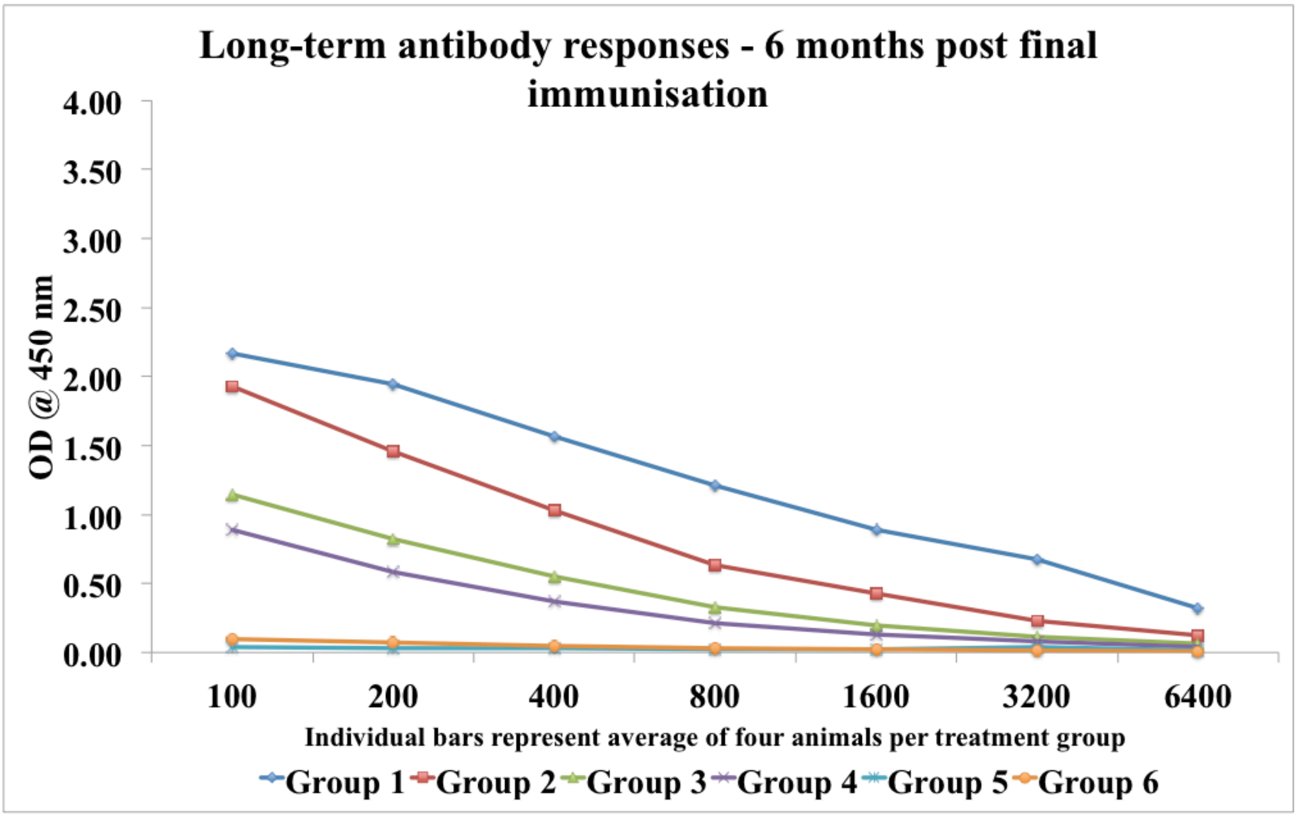

Long-term antibody responses - 6 months post final immunisation
OD @ 450 nm
4.00
3.50
3.00
2.50
2.00
1.50
1.00
0.50
0.00
100
200
400
800
1600
3200
6400
Individual bars represent average of four animals per treatment group
Group 1
Group 2
Group 3
Group 4
Group 5
Group 6
